# The antioxidant and antiproliferative activities of methanolic extracts from Njavara rice bran

**DOI:** 10.1186/1472-6882-10-4

**Published:** 2010-01-28

**Authors:** Akiri SVC Rao, Sareddy G Reddy, Phanithi P Babu, Attipalli R Reddy

**Affiliations:** 1 Department of Plant Sciences, School of Life Sciences, University of Hyderabad, Hyderabad - 500 046, India; 2 Department of Biotechnology, School of Life Sciences, University of Hyderabad, Hyderabad - 500 046, India

## Abstract

**Background:**

Free radical-induced oxidative stress is the root cause for many human diseases. Naturally occurring antioxidant supplements from plants are vital to counter the oxidative damage in cells. The main objective of the present study was to characterize the antioxidant and antiproliferative potential of rice bran extracted from an important Indian rice variety, Njavara and to compare the same with two commercially available basmati rice varieties: Vasumathi, Yamini and a non medicinal variety, Jyothi.

**Methods:**

Methanolic extracts of rice bran from four varieties; Vasumathi, Yamini, Jyothi and Njavara were used to study their total phenolic and flavonoid contents, *in vitro *antioxidant activities including total antioxidant activity, scavenging of nitric oxide and 1,1-Diphenyl-2-picrylhydrazyl (DPPH) radical, reducing power and cytotoxic activity in C6 glioma cells. Correlation coefficient and regression analysis were done by using Sigmastat version 3.1 and Stata statistical package respectively.

**Results:**

Rice bran methanolic extract from Njavara showed the highest antioxidant and cell cytotoxic properties compared to the other three rice varieties. IC_50 _values for scavenging DPPH and nitric oxide were in the range of 30.85-87.72 μg/ml and 52.25-107.18 μg/ml respectively. Total antioxidant activity and reducing power were increased with increasing amounts of the extract. Total phenolic and flavonoid contents were in the range of 3.2-12.4 mg gallic acid-equivalent (GAE)/g bran and 1.68-8.5 mg quercetin-equivalent (QEE)/g bran respectively. IC_50 _values of cytotoxic assay (MTT assay) were 17.53-57.78 μg/ml. Correlation coefficient and regression analysis of phenolic content with DPPH and NO scavenging, MTT (-[4,5-dimethylthiazol-2-yl]-2,5-diphenyl tetrazolium bromide) assay, total antioxidant assay and reducing power showed a highly significant correlation coefficient values (96-99%) and regression values (91-98%).

**Conclusion:**

The results of the present study show that the crude methanolic extract from Njavara rice bran contains significantly high polyphenolic compounds with superior antioxidant activity as evidenced by scavenging of free radicals including DPPH and NO. Njavara extracts also showed highest reducing power activity, anti-proliferative property in C6 glioma cells. In conclusion, it is conceivable that the Njavara rice variety could be exploited as one of the potential sources for plant - based pharmaceutical products.

## Background

Rice bran is a rich source of natural antioxidants which can be used as free radical scavengers. It is widely recognized that many of the today's diseases are due to the oxidative stress that results from an imbalance between formation and neutralization of pro-oxidants [1,2]. Cells have developed antioxidant mechanisms to quench the free radicals but when the generation of free radicals exceeds the scavenging capacity of the cell, the excess free radicals seek stability through electron pairing with biological macromolecules such as proteins, lipids and DNA in healthy human cells resulting the induction of lipid peroxidation which leads to cancer, atherosclerosis, cardiovascular diseases, ageing and inflammatory diseases [3-7]. The free radicals are known to be scavenged by synthetic antioxidants, but due to their adverse side effects leading to carcinogenicity; search for effective and natural antioxidants has become crucial [8,9]. Rice bran is a by-product of rice milling which contains a significant amount of natural phytochemicals including sterols, higher alcohols, gamma-oryzanol, tocopherols, tocotrienols and phenolic compounds [10-12]. These bioactive molecules have known to reduce serum cholesterol, decrease the incidence of atherosclerosis and also have antitumor properties [13-16].

Rice is a staple food for more than three billion people in the world. The ayurvedic treatise records show the existence of several medicinal rice varieties in India. Njavara is one of such important Indian medicinal rice variety, grown in Southern India and is used mainly for ayurvedic treatments [17,18]. It is regarded as a special rice variety with beneficial properties for the circulatory, respiratory, digestive and nervous systems according to the Indian indigenous system of medicine or ayurveda [17].

Njavarakizhi and Navaratheppu are the two major treatments in ayurveda for arthritis, paralysis, neurological disorders, degeneration of muscles and tuberculosis. In addition to various medicinal properties, Njavara gruel is also included in the diet for developing immunity. Considered as gold among paddy varieties, recently Deepa et al., [17] reported certain nutritional properties of Njavara while Simi and Abraham studied the physiochemical, rheological and thermal properties of Njavara rice starch [18]. However, no scientific data are available on the free radical scavenging and cell cytotoxic properties of the Njavara rice bran extract. Our main objective in this investigation was to demonstrate antioxidative and radical scavenging properties of Njavara rice bran methanolic extract. Njavara rice bran properties were compared with commercially available two Indian basmati varieties: Vasumathi, Yamini and a non medicinal variety, Jyothi.

## Methods

### Chemicals

Folin-Ciocalteus's phenol reagent, sodium carbonate, gallic acid (GA), quercetin (QE), FeCl_3_, NaNO_2_, 1,1-Diphenyl-2-picrylhydrazyl (DPPH), ascorbic acid were purchased from Sigma Chemical Co. (St. Louis, MO, USA). Sodium nitro preside (SNP), α-napthyl-ethylenediamine, potassium ferricyanide, trichloroacetic acid (TCA), ammonium molybdate, 3-[4,5-dimethylthiazol-2-yl]-2,5-diphenyl tetrazolium bromide (MTT), dimethyl sulphoxide (DMSO) were purchased from Merck Chemical Supplies (Damstadt, Germany). All the chemicals used including the solvents, were of analytical grade.

### Plant material

Seeds of Njavara variety were obtained from Kerala Agricultural University, Kerala, Vasumathi, Yamini and Jyothi varieties were obtained from Directorate of Rice Research, Hyderabad; Central Soil Salinity Research Institute, Karnal and National Seed Corporation, Warangal respectively.

### Preparation and stabilization of rice bran

Rice bran from the four varieties was obtained by milling rice grain in a local grinding mill, followed by sieving to separate grain from bran. Stabilization of rice bran was done by heating the bran in microwave oven at 850W and with 2450 MHz. The microwave chamber was preheated to 100% power for 3 min. The moisture content of the raw rice bran was adjusted to 21% by adding deionized water [19]. The sample was heated for 3 min at 100% power. The temperature of the sample after heating in the microwave was 107 ± 2°C. The sample was allowed to cool to room temperature and stored in an ultralow freezer (-80°C) until further analysis.

### Preparation of rice bran extract

Rice bran extract was prepared according to a modified method of Choi et al., [20]. Rice bran powder (5 g) was extracted thrice with 30 ml methanol for 3 h in an electrical shaker at 40°C. The extracts were filtered through Whatman No.2 filter paper and evaporated under vaccum using a rotary evaporator (Heidolph, Germany). The residual crude methanolic rice bran extract was weighed and dissolved in dimethyl sulphoxide (DMSO), and filtered through a 0.45 μm of Nylon membrane filter and stored at -20°C until further analysis.

### Determination of total phenolic content

The total phenolic content of the bran extracts was determined using the Folin- Ciocalteu reagent [21]. The reaction mixture contained: 200 μl of diluted rice bran extract, 800 μl of freshly prepared diluted Folin Ciocalteu reagent and 2 ml of 7.5% sodium carbonate. The final mixture was diluted to 7 ml with deionized water. Mixtures were kept in dark at ambient conditions for 2 h to complete the reaction. The absorbance at 765 nm was measured. Gallic acid was used as standard and the results were expressed as mg gallic acid (GAE)/g bran.

### Determination of total flavonoid content

Total flavonoid content was determined using aluminium chloride (AlCl_3_) according to a known method [22] using quercetin as a standard. The plant extract (0.1 ml) was added to 0.3 ml distilled water followed by 5% NaNO_2 _(0.03 ml). After 5 min at 25°C, AlCl_3 _(0.03 ml, 10%) was added. After further 5 min, the reaction mixture was treated with 0.2 ml of 1 mM NaOH. Finally, the reaction mixture was diluted to 1 ml with water and the absorbance was measured at 510 nm. The results were expressed as mg quercetin (QE)/g bran.

### Determination of reducing power

The reducing power of rice bran extract was measured according to the method described by Yen and Duh [23] with some modifications. Various concentrations (100, 200, 300, 400 and 500 μg) of rice bran extract were mixed with 2.5 ml of 0.2 M sodium phosphate buffer (pH 6.6). The dilute sample was then mixed with 5.0 ml of 1% potassium ferricyanide and the mixture was incubated at 50°C for 20 min. 5.0 ml of 10% trichloroacetic acid was added to the mixture, which was then centrifuged at 3000 rpm for 10 min. 5.0 ml of the supernatant was mixed with 5.0 ml of distilled water and 1.0 ml of ferric chloride (1%). The absorbance was measured at 700 nm. Ascorbic acid was used for comparison.

### Determination of total antioxidant activity

For total antioxidant assay various concentrations (20, 40, 60, 80 and 100 μg) of rice bran extract were mixed with 1 ml of the reagent solution (0.6 M sulfuric acid, 28 mM sodium phosphate and 4 mM ammonium molybdate). The reaction mixture was incubated in a water bath at 95°C for 90 min. After cooling to room temperature, the absorbance was measured at 695 nm [24]. Ascorbic acid was used for comparison.

### Measurement of nitric oxide scavenging ability

Nitric oxide scavenging activity was determined according to Griess Illosvoy reaction [25]. The reaction mixture contained: 10 mM SNP in 0.5 M phosphate buffer, pH 7.4, and various doses (50-250 μg/ml) of the test solution in a final volume of 3 ml. After incubation for 60 min at 37°C, Griess reagent (α-napthyl-ethylenediamine 0.1% in water and sulphanilic acid 1% in H_3_PO_4 _5%) was added. The pink chromophore generated during diazotization of nitrite ions with sulphanilamide and subsequent coupling with α-napthyl-ethylenediamine was measured spectrophotometrically at 540 nm. Ascorbic acid was used as a positive control. Nitric oxide scavenging ability (%) was calculated by using the formula:

### Determination of DPPH·scavenging assay

DPPH radical scavenging activity of rice bran extract was determined according to the method reported by Blois [26] with slight modifications. An aliquot of 0.5 ml of sample solution in methanol was mixed with 2.5 ml of 0.5 mM methanolic solution of DPPH. The mixture was shaken vigorously and incubated for 37 min in the dark at room temperature. The absorbance was measured at 517 nm using UV-vis spectrophotometer. Ascorbic acid was used as a positive control. DPPH free radical scavenging ability (%) was calculated by using the formula:

### In vitro cell cytotoxicity activity (MTT assay)

The cell cytotoxicity of methanolic rice bran extracts against the C6 glioma cells was determined by MTT assay [27]. Cells were seeded into 96-well plate at 1 × 10^4 ^cells/well density and treated with different concentrations of rice bran extracts for 48 h. After 48 h MTT was added to each well and solubilized the formazan crystals by DMSO. Then absorbance was measured at 570 nm in a microplate ELISA reader. All experiments were performed in triplicate.

### Statistical analysis

The data were subjected to correlation coefficient by using Sigmastat version 3.1 statistical analysis software. The correlation of the data was determined by Pearson's test and Stata statistical package was used for regression analysis. P < 0.05 was considered as statistically significant.

## Results

### Total phenolic content (TPC)

The total phenolic content (TPC) was expressed as gallic acid equivalents (Table 1). Significant differences were observed for TPC among the four rice varieties. TPC was in the range of 3.27-12.4 mg GAE/g bran. Highest TPC was observed in Njavara followed by Jyothi. Yamini and Vasumathi showed nearly equal TPC content which were in low quantities compared to Njavara.

**Table 1:** Total phenolic and flavonoid contents from methanolic rice bran extracts.

Rice Variety	Total phenolic extract (mg GAE/g rice bran)	Total flavonoid content (mg QE/g rice bran)
Vasumathi	3.31 ± 0.3	1.68 ± 0.032

Yamini	4.23 ± 0.4	2.57 ± 0.041

Jyothi	9.44 ± 0.2	5.33 ± 0.072

Njavara	12.72 ± 0.6	8.51 ± 0.053

Values are of three experiments ± SE

### Total flavonoid content (TFC)

Total flavonoid content of the methanolic extract was significantly high in Njavara compared to the other three varieties as recorded in quercetin equivalents (Table 1). TFC of Vasumathi, Yamini, Jyothi, and Njavara were 1.68, 2.57, 5.33 and 8.5 mg QEE/g bran respectively.

### Total antioxidant activity (TAA)

**Figure 1:**
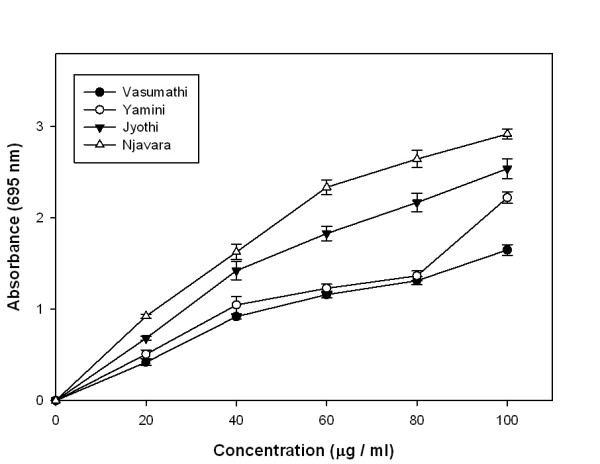
**Total antioxidant activity**. TAA carried out with different concentrations of methanolic rice bran extracts from four rice varieties. Results represent means ± standard deviation (n = 3).

Total antioxidant activity of the rice bran extracts increased with the increasing concentration of the extracts and a significant change was observed at 0.02 to 0.1 mg/ml concentration of the extract (Figure 1). At 0.1 mg/ml of the methanolic rice bran extracts, the absorbance values of Vasumathi, Yamini, Jyothi and Njavara were 1.65, 2.21, 2.52 and 2.93 respectively. However, the total antioxidant activities of all the rice bran extracts were less than that of the positive control ascorbate (2.5-40 μg showed an absorbance 0.117-2.0).

### Reducing power (RP) assay

**Figure 2:**
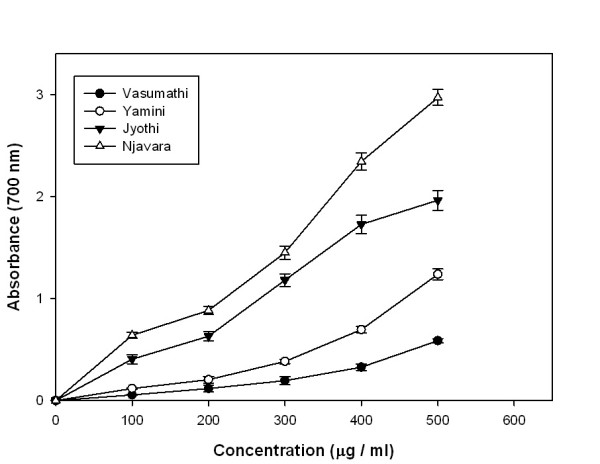
**Reducing power**. RP of different concentrations of methanolic rice bran extracts from four rice varieties. Results represent means ± standard deviation (n = 3).

The methanolic extracts from all the samples had shown considerable amount of reducing activity. The reducing power of the rice bran extracts increased with the increasing concentration and a significant change was observed at 0.1 to 0.5 mg/ml concentration of the rice extract (Figure 2). 0.5 mg/ml of the methanolic rice bran extracts showed absorbance values of 0.59, 1.04, 1.93 and 2.98 corresponding to Vasumathi, Yamini, Jyothi and Njavara were respectively. While 5-25 μg of the positive control ascorbate showed an absorbance 0.06-0.28.

### DPPH radical scavenging activity

**Figure 3:**
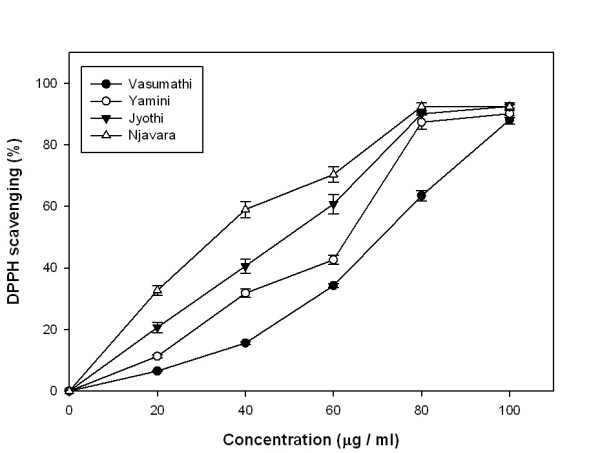
**DPPH radical scavenging activity (%)**. DPPH scavenging activity of different concentrations of methanolic rice bran extracts from four rice varieties. Results represent means ± standard deviation (n = 3).

Free radical scavenging activities of the rice bran methanolic extracts were assessed by the DPPH assay. Figure 3 illustrates a significant decrease in the concentration of DPPH radical due to scavenging ability of the rice bran. The results show that Njavara had the highest DPPH scavenging activity with an IC_50 _value of 30.85 μg/ml. IC_50 _values of the other three varieties were 48.88, 70.58 and 87.72 μg/ml for Jyothi, Yamini and Vasumathi respectively. IC_50 _value of the positive control ascorbic acid was 3.2 μg/ml.

### Nitic oxide (NO) scavenging

**Figure 4:**
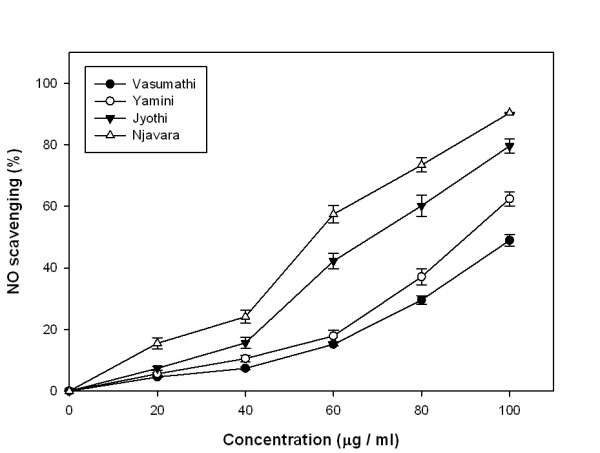
**Nitric oxide scavenging activity (%)**. NO scavenging activity in the methanolic rice bran extracts from four rice cultivars. Results represent means ± standard deviation (n = 3).

Njavara extract showed the highest nitric-oxide scavenging activity compared to the other three rice bran methanolic extracts in a moderate dose dependent inhibition of nitric oxide with an IC_50 _value of 52.25 μg/ml. IC_50 _values of Jyothi, Yamini and Vasumathi were 71.41, 107.18 and 102.48 μg/ml respectively (Figure 4). Ascorbic acid was used as a reference compound and its IC_50 _value is 4.6 μg/ml.

### In vitro cytotoxic activity (MTT assay)

**Figure 5:**
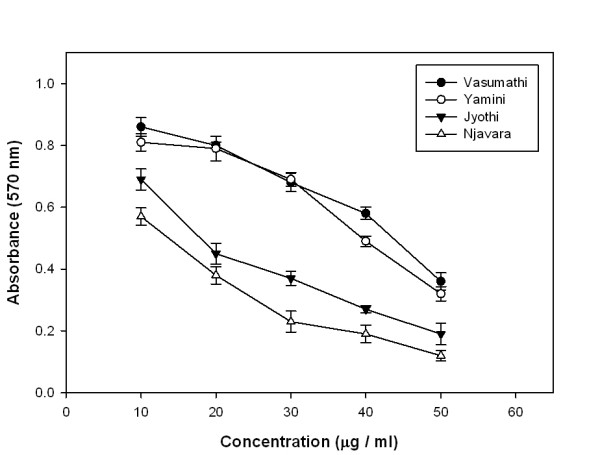
**Cell cytotoxicity assay**. MTT assay with different concentrations of methanolic rice bran extracts from four rice cultivars. Results represent means ± standard deviation (n = 3).

The cytotoxic activity of the methanolic extracts from rice bran against C6 glioma cells were shown in figure 5. IC_50 _values of the methanolic rice bran extracts of Vasumathi, Yamini, Jyothi, and Njavara were 57.78, 44.83, 25.52 and 17.53 μg/ml respectively.

### Statistical analysis

Table 2 shows highly positive significant correlation coefficient values for phenolic content with DPPH and NO scavenging; TAA and RP. A negative significant correlation coefficient was observed for phenolic content with MTT in the four rice varieties. Table 3 shows the regression analysis for the four rice varieties. The slope of Vasumathi showed higher significant value for phenolic content with DPPH scavenging (6.44) and TAA (0.087) compared to the other varieties. It also showed a higher slope (3.39) with NO but less significant over Jyothi. Yamini had a higher significant slope (0.012) for phenolic content with RP. Njavara had higher negative significant slope (-0.017) for phenolic content with MTT.

**Table 2:** Correlation of phenolic contents from the methanolic bran extracts of different rice varieties with DPPH radical scavenging, Nitric oxide (NO) scavenging, Total Antioxidant Assay (TAA), MTT assay and Reducing Power (RP).

	TPC vs DPPH scavenging	TPC vs NO scavenging	TPC vs TAA	TPC vs MTT assay	TPC vs RP
**Vasumathi**					
R value	0.984	0.957	0.981	-0.976	0.955
P value	0.00247	0.0105	0.00313	0.00451	0.0114

**Yamini**					
R value	0.968	0.952	0.953	-0.963	0.950
P value	0.00689	0.0125	0.0123	0.00862	0.0132

**Jyothi**					
R value	0.981	0.992	0.985	-0.969	0.989
P value	0.00325	0.000938	0.00217	0.00655	0.00142

**Njavara**					
R value	0.964	0.987	0.977	-0.961	0.992
P value	0.00826	0.00186	0.00427	0.00923	0.000800

In all the experiments number of samples (n) = 5. The pair(s) of variables with positive correlation coefficients and P values below 0.050 tend to increase together. For the pairs with negative correlation coefficients and P values below 0.050, one variable tends to decrease while the other increases. For pairs with P values greater than 0.050, there is no significant relationship between the two variables.

**Table 3:** Regression analysis of polyphenolic content from the methanolic bran extracts of different rice varieties with DPPH radical scavenging, Nitric oxide (NO) scavenging, Total Antioxidant Assay (TAA), MTT assay and Reducing Power (RP).

	TPC vs DPPH scavenging	TPC vs NO scavenging	TPC vs TAA	TPC vs MTT assay	TPC vs RP
**Vasumathi**					
R^2 ^value	0.9678	0.9163	0.9624	0.9521	0.9120
Slope	6.435366	3.385976	0.0870031	-0.0743902	0.0077537
P value	0.0025	0.0105	0.0031	0.0045	0.0114
Growth rate	66.18	61.46	30.93	-20.63	57.52

**Yamini**					
R^2 ^value	0.9367	0.9067	0.9074	0.9267	0.9029
Slope	4.82466	3.172851	0.0123	-0.0579186	0.0123534
P value	0.0069	0.0125	0.0849	0.0086	0.0132
Growth rate	51.68	60.85	32.24	-23.35	59.25

**Jyothi**					
R^2 ^value	0.9615	0.9831	0.9705	0.9388	0.9777
Slope	2.045763	2.002225	0.0472744	-0.025	0.0089229
P value	0.0033	0.0009	0.0022	0.0066	0.0014
Growth rate	37.95	61.04	30.51	-30.9	41.61

**Njavara**					
R^2 ^value	0.9287	0.9733	0.9538	0.9233	0.9848
Slope	1.199922	1.561774	0.039325	-0.0171115	0.0101612
P value	0.0083	0.0019	0.0043	0.0092	0.0008
Growth rate	25.28	46.37	27.86	-38.09	46.91

R^2 ^values are the regression coefficients by the least -- squared fits and P = probability values. In all the experiments number of observations (n) = 5.

## Discussion

Total antioxidant activity of the rice bran methanolic extracts increased with increasing concentration of the extracts indicating the potential of rice bran extracts as antioxidants. Relatively high total antioxidant activity in the Njavara rice bran compared to the other samples showed a significant correlation with polyphenolic contents (Table 2) suggesting the importance of polyphenolics as potential antioxidant biomolecules. Reducing power has been used to evaluate the ability of natural antioxidants in the rice bran extracts to donate electrons [28]. Njavara had relatively higher reducing power than other samples, indicating a significantly higher correlation with polyphenolic content (Table 2 and 3). The results indicate that rice bran methanolic extracts are capable of donating electrons which can react with free radicals to convert them as more stable products and strongly inhibiting radical chain reaction.

DPPH radical scavenging is considered a good *in vitro *model widely used to assess antioxidant efficacy within a very short time. In its radical form, DPPH^· ^has disappears on reduction by an antioxidant compound or a radical species to become a stable diamagnetic molecule resulting the colour change from purple to yellow, which could be taken as an indication of the hydrogen donating ability of the tested samples [29,30]. DPPH radical scavenging abilities of the rice bran extracts were significantly lower than those of ascorbic acid. However, all the rice varieties in our study exhibited appreciable scavenging activity and there was a significant correlation between DPPH radical scavenging activity and polyphenolic content. IC_50 _value of Njavara indicates that it has a highest proton donating ability among the four tested rice varieties (Figure 3). The results indicate that the extracts with their proton-donating ability, could serve as free radical inhibitors or scavengers, acting possibly as primary antioxidants [29]. Our data demonstrate that the rice bran extracts inhibit nitrite formation by directly competing with oxygen in the reaction with nitric oxide [31]. The present study also proved that the rice bran methanolic extracts have potent nitric oxide scavenging activities and Njavara had the highest nitric oxide scavenging activity compared to the other three varieties. These oxy-radicals are to be toxic to the tissues and are responsible for various inflammatory responses and carcinomas. Excess nitric oxide which is known to accumulate in the acidic environment of stomach reacts with oxygen to form nitrite ions and induce mutagenic reactions [32]. It has recently been reported that phenolic compounds have a greater nitrite scavenging activity in environments with low pH [33]. The results of this study show that the bran from all the rice varieties contain a notable antiproliferative activity and the biomolecules from the rice bran, particularly from the Njavara variety could be exploited for the potential use in pharmaceutical formulations.

Our results indicate that rice bran extracts from all the four varieties contain significant amounts of flavonoids and Njavara possessed the highest flavonoid levels (Table 1) Flavonoids are the most ubiquitous groups of plant secondary metabolites [34]. This class of compounds have good antioxidant potential and their effects on human nutrition and health are considerable. Flavonoids have been widely used in cancer treatments, coronary heart diseases, gastrointestinal ulcers and rheumatic diseases [35]. Polyphenols are the major plant compounds with potential antioxidant activity. This activity is believed to be mainly due to their redox properties, which play an important role in adsorbing and neutralizing free radicals, quenching singlet and triplet oxygen, or decomposing peroxides [36-39]. In the present study, antiradical efficiency in the rice bran extractswas highly positively correlated with total phenolic contents (Table 2). Our data also indicate that polyphenols are important components in rice bran methanolic extracts which could be exploited for their use in free radical scavenging activity.

### Statistical analysis

Regression analysis of phenolic content with DPPH and NO scavenging, TAA and RP showed higher slope values in Vasumathi compared to the Njavara. Higher slope values in Vasumathi can be explained by the higher percentage of growth rate compared to Njavara. Minimum IC_50 _values of Njavara has been predicted to be responsible for the lesser growth rate compared to Vasumathi resulting lesser slope values. Correlation coefficient and regression analysis showed that total phenolic content was responsible for antiradical efficiency in the rice bran extracts.

## Conclusion

Our results strongly suggest that medicinal rice plants can be promising sources of potential antioxidants and anticancer activity. The present results will form the basis for selection of Njavara species for further investigation in the potential drug discovery of new natural bioactive compounds. Njavara is a good choice for the plant scientists to develop new rice cultivars with high bioactive compounds with high nutritive value. This is the first report on the antiradical efficiency of the Indian medicinal rice, Njavara. Studies aimed at isolation and structural elucidation of the anticancer and antioxidative active constituents from Njavara are in progress.

## Competing interests

The authors declare that they have no competing interests.

## Authors' contributions

ASVCR: Performed the study and prepared the manuscript; SGR: Provided assistance in the investigation on cell lines; PPB: Provided cell lines and supervised the study on cell lines; ARR: Supervised the work, provided the grants for the study, evaluated the data, corrected the manuscript for publication and coordinated the study. All the authors have approved the final manuscript.

## Pre-publication history

The pre-publication history for this paper can be accessed here:

http://www.biomedcentral.com/1472-6882/10/4/prepub
